# Pulmonary function testing in patients with liver cirrhosis (Review)

**DOI:** 10.3892/mi.2023.96

**Published:** 2023-07-06

**Authors:** Vasiliki Epameinondas Georgakopoulou, Stavroula Asimakopoulou, Evangelos Cholongitas

**Affiliations:** 1 Department of Infectious Diseases and COVID-19 Unit, Laiko General Hospital, Medical School, National and Kapodistrian University of Athens, 11527 Athens, Greece; 2 First Department of Internal Medicine, Laiko General Hospital, Medical School, National and Kapodistrian University of Athens, 11527 Athens, Greece

**Keywords:** liver cirrhosis, spirometry, lung function, diffusion capacity, hepatopulmonary syndrome

## Abstract

Liver cirrhosis is a common long-term outcome of chronic hepatic inflammation. Patients with liver cirrhosis may also have pulmonary complications. There are several reasons for pulmonary dysfunction in liver cirrhosis, including intrinsic cardiopulmonary dysfunction unrelated to liver disease and specific disorders related to the presence of liver cirrhosis and/or portal hypertension. The most prevalent and clinically significant pulmonary complications are hepatic hydrothorax, hepatopulmonary syndrome, spontaneous pulmonary empyema and portopulmonary hypertension. Pulmonary function tests (PFTs) have traditionally been used to assess the lung function of patients with liver cirrhosis. To the best of our knowledge, the present review is the first to detail all types of PFTs performed in patients with liver cirrhosis and discuss their clinical significance. Patients with liver cirrhosis have reduced values of spirometric parameters, diffusion capacity for carbon monoxide (DLCO), lung volumes, maximal inspiratory pressure and maximal expiratory pressure. Furthermore, they have a higher closing volume, a greater airway occlusion pressure 0.1 sec after the onset of inspiratory flow and greater exhaled nitric oxide values. In order to improve pulmonary function, patients with ascites may require therapeutic paracentesis. Such findings should be considered when evaluating individuals with liver disease, particularly those who may require surgery. Poor lung function, particularly restrictive lung disease, can have an impact on post-transplant outcomes, such as ventilator time, length of hospital duration and post-operative pulmonary complications; thus, the transplant care team needs to be aware of its prevalence and relevance.

## 1. Introduction

Cirrhosis is a common long-term result of persistent hepatic inflammation. Liver cirrhosis can be caused by various toxic, metabolic, infectious, or autoimmune conditions such as alcoholism, non-alcoholic fatty liver disease (NAFLD), autoimmune hepatitis, viral hepatitis, primary biliary cholangitis (PBC), and primary sclerosing cholangitis, as well as a variety of metabolic disorders such as Wilson's disease, hemochromatosis and alpha-1-antitrypsin deficiency (1). The consequences in the function and anatomy of the liver include: i) Hepatic insufficiency with reduced synthesis and impaired metabolic functions; ii) the development of intrahepatic portosystemic shunts between portal vessels and hepatic veins in a sequential formation; and iii) portal hypertension (2,3). Decompensated cirrhosis occurs when clinically relevant complications and sequelae of portal hypertension (e.g., ascites, variceal bleeding, hepatorenal syndrome) occur along with the deterioration of liver function (e.g., decreased formation of coagulation factors, insufficient degradation of ammonia resulting in hepatic encephalopathy) (2,3).

Pulmonary complications may develop in patients with or without liver decompensation (4). Up to 70% of patients suffering from liver cirrhosis who are evaluated for liver transplantation complain of dyspnea (5). In addition, as many as 45% of patients with chronic liver disease who participated in screening studies had abnormal arterial blood gas reports (6). These complications should be distinguished from primary lung disorders, such as chronic obstructive pulmonary disease (COPD), which can occur in patients with liver diseases, but are not pathogenically linked to liver cirrhosis (4).

Several causes of pulmonary dysfunction in liver cirrhosis have been recognized, including intrinsic cardiopulmonary disorders unrelated to liver disease and unique disorders related to the presence of liver disease and/or portal hypertension (7). Hepatic hydrothorax, hepatopulmonary syndrome (HPS), spontaneous pulmonary empyema and portopulmonary hypertension are the most common and clinically important pulmonary consequences (4).

Conventional pulmonary function tests (PFTs) have been used to measure the pulmonary function of patients with liver cirrhosis (8). PFTs are a critical diagnostic and monitoring modality for individuals with respiratory disease. They provide vital information regarding the function of the large and small airways, the lung parenchyma, as well as the size and integrity of the pulmonary capillary bed. Although they do not provide a diagnosis in and of themselves, diverse patterns of anomalies are detected in various respiratory disorders, which aid in diagnosis (9,10).

Pulmonary alterations may be present in approximately one third of patients with decompensated liver cirrhosis, leading to a decrease in arterial oxygen saturation and sometimes to cyanosis (11). Additionally, abnormalities in PFTs and impaired gas exchange may develop in as many as 45-50% of patients with liver cirrhosis (12). Certain pulmonary functions may be impaired in chronic liver disease. In general, airway obstruction, impairment in diffusion capacity for carbon monoxide (DLCO) and a reduction in total lung capacity (TLC) indicating a restrictive type of abnormality are manifested, leading to the deterioration of gas exchange and hypoxemia (11,13). Furthermore, apart from traditional PFTs, additional PFTs, such as the calculation of the airway occlusion pressure 0.1 sec after the onset of inspiratory flow (P0.1), which is a useful tool for the evaluation of respiratory motor output (14), have been conducted in individuals with liver cirrhosis and have been associated with disease severity (15).

To the best of our knowledge, the present review is the first to describe all the types of PFTs that have been conducted in patients with liver cirrhosis and to discuss their clinical significance.

## 2. Spirometry

Spirometry determines the maximum amount of air that a patient can inhale and exhale while exerting maximum effort, calculating volume or flow as a function of time. The most frequent measurements include the forced vital capacity (FVC), which quantifies the amount of air exhaled during a full and vigorous expiration, the forced expiratory volume in one second (FEV1) and peak expiratory flow rate (PEFR) (16). The mean forced expired flow when lung volume declines from 75 to 25% of vital capacity [forced expiratory flow between 25 and 75% of vital capacity (FEF25-75%)] is another variable that may be assessed during the FVC maneuver and is linked to small airway impairment (17).

Variations in the values of FEV1, FVC, PEFR and FEF25-75% have been described in patients with liver cirrhosis (13,15,18-40). More specifically, some researchers (13,15,18,27,32) have reported obstructive dysfunction, while others (19,21,23,25,26,31,33,38) reported obstructive and/or restrictive ventilatory abnormalities, and some researchers (11,20,22-24,28-30,32,34-37,39,40) found isolated declines in the absolute values of FEV1, FVC, PEFR and/or FEF25-75%.

Notably, in some studies, these dysfunctions were shown to be associated with the severity of liver cirrhosis, as assessed by various scores, such as the Child Pugh Score and the Model for End-Stage Liver Disease (MELD) score, clinical characteristics such as ascites and laboratory parameters such as albumin (11,13,19,20,23,26-28,32,33,35,36,40). Some studies (19,20,23,33,35) have found a significant association between decreasing PEFR, FVC, FEV1 and FEF25-75% values, and increasing ascites. In addition, a statistically significant positive correlation between FEV1 and serum albumin, and between FVC and serum albumin has been described in patients with liver cirrhosis (32). Moreover, FEV1 and FVC values have been shown to be positively associated with the 6-min walking test during the pre-transplant evaluation of patients with cirrhosis (36).

In addition, a significant reduction in FEF25-75% values has been observed in patients with esophageal varices, while an obstructive dysfunction has been detected in patients with alcoholic cirrhosis (19).

Of note, a restrictive spirometric alteration has been statistically associated with a higher Child Pugh Score, a higher MELD score, the presence of pleural effusions, encephalopathy, ascites, hepatic hydrothorax, lower albumin levels, the presence of hyperbilirubinemia and worse exercise capacity, quality of life, and survival rates (19,33). Moreover, a restrictive spirometric pattern has been associated with tense ascites (26).

The Child-Pugh score has been found to be negatively associated with FEV1 (11,28), FVC (32) and FEV1/FVC values (13), while the Glasgow Alcoholic Hepatitis Scale (GAHS) has been negatively associated with FEV1/FVC values (27).

Of note, in a study evaluating the presence of non-specific impairment of lung functions (NILF), defined as the observation of any two of three following criteria: i) FVC <80% of predicted; ii) FEV1 <80% of predicted; iii) FEV1/FVC ≥70, NILF was statistically associated with the female sex and with increasing FibroScan scores (40).

## 3. Diffusing capacity for carbon monoxide

DLCO testing is used to identify patients who have exertional dyspnea, spirometric obstruction or restriction, interstitial lung disease, pulmonary vascular disorders, occupational pulmonary diseases and/or pulmonary side effects of radiation or medications (41). A low DLCO is value the most common lung function alteration identified in chronic liver disease. Diffusion capacity is the volume of any gas that diffuses across the alveolo-capillary membrane in one unit of time (1 min) with a certain pressure gradient (1 mmHg). DLCO/VA, on the other hand, is the diffusion capacity of one liter of lung volume (23).

Decreased DLCO and DLCO/VA values have been reported in patients with liver cirrhosis (6,21,23,24,25,34,38,39,42-44). Similar to spirometric parameters, in some studies, alterations in DLCO and DLCO/VA values have been found to be associated with the severity of liver cirrhosis as assessed by various scores, clinical features and laboratory data (23,24,27,38,43,44). More specifically, both DLCO and DLCO/VA have been shown to negatively correlate with the Child-Pugh score (23,24,27), while DLCO/VA has also been shown to exhibit a negative correlation with the MELD score (43). In addition, DLCO has been found to exhibit a significant positive correlation with serum albumin and cholinesterase levels (24), and a significant negative correlation with esophageal varices and ascites (43). Moreover, DLCO and DLCO/VA values have demonstrated an inverse linear correlation with the heart/liver ratio (H/L) in thallium-201 per rectum scintigraphy, which indirectly indicates a portosystemic shunt (44). Of note, DLCO has been described as a significant predictor of ventilator time and both intensive care unit (ICU) and hospital length of stay (LOS) following liver transplantation (38).

## 4. Lung volumes

TLC and residual volume (RV), which indicates the amount of air left in the respiratory tract at the end of a maximal expiration, can be estimated using either gas dilution or whole-body plethysmography (45). The air volume that remains in the respiratory system following a normal exhalation is referred to as functional residual capacity (FRC). The FRC increases as lung volumes increase (46).

The RV, FRC and TLC values have been found to be elevated, decreased, or normal in patients with liver cirrhosis. More specifically, the RV and TLC values have been observed to be increased in patients with liver cirrhosis, indicating air trapping (47), or to be normal (18). However, the majority of studies have demonstrated that lung volumes are decreased in patients with liver cirrhosis (20,24,27,35,38). As regards the association between lung volumes and the severity of liver cirrhosis and clinicolaboratory characteristics, TLC has been shown to exhibit a significant positive correlation with serum albumin levels (24) and a significant negative correlation with the presence of ascites (35). Furthermore, TLC has been found to exhibit a significant negative correlation with the GAHS scale (27), and both TLC and RV have been found to be significant predictors of ventilator time and both ICU and hospital LOS following liver transplantation (38).

## 5. Single breath gas washout

Small airway closure with increased lung volumes is a feature of various respiratory disorders, including asthma and chronic obstructive pulmonary disease, and identifying this alteration may aid in the early detection of respiratory impairment (48).

The reference method for investigating airway closure is the single breath gas washout (SBW), commonly nitrogen. The exhaled used gas concentration vs. exhaled volume trace after a vital capacity inhalation of a used gas-free gas mixture exhibits an initial rapid increase (phase II) to a slow-rising alveolar plateau (phase III), and then an abrupt change in the slope that signals the beginning of phase IV. The closing volume (CV) represents the volume at the start of phase IV (49).

The CV has been reported to be increased in patients with liver cirrhosis, suggesting that the narrowing or closure in small airways may develop in these patients, while the association of these alterations in CV with disease severity remains unclear (18,47,50).

## 6. Airway occlusion pressure 0.1 sec after the onset of inspiratory flow

Airway occlusion pressure 0.1 sec after the onset of inspiratory flow (P0.1) is the negative airway pressure developed during the first 100 msec of an obstructed inspiration. P0.1 is a measure for the neuromuscular activation of the respiratory system, which is a key predictor of breathing functions. It has been demonstrated to be a good predictive indicator of efficient mechanical ventilation weaning. The standard P0.1 measuring procedures rely on occluding the inspiration for >100 msec (51).

Increased values of P0.1 have been observed in patients with liver cirrhosis (15). Moreover, P0.1 has been shown to positively correlate with FEV1/FVC. In addition, P0.1 has been found to positively correlate with the MELD score, indicating the presence of abnormal increased respiratory drive in these patients (15).

## 7. Measurement of maximal inspiratory pressure and maximal expiratory pressure

Maximal inspiratory pressure (MIP) and maximal expiratory pressure (MEP) are non-invasive, simple and practical indicators of respiratory muscle strength at the mouth (52). MIP and MEP values have been described to be affected in patients with liver cirrhosis (35,53-55). MIP and MEP values have been found to positively correlate with the presence of ascites and with the MELD score (35). MIP and MEP values have also been shown to correlate with the modified medical research council dyspnea scale score in patients with liver cirrhosis (35). In addition, MIP and MEP values have been reported to be lower in patients with liver cirrhosis due to alcohol consumption compared to those with liver cirrhosis due to hepatitis B virus and hepatitis C virus (54). Of note, MIP has been reported as a predictive indicator of mortality in patients with liver cirrhosis in a previous study (54).

## 8. Exhaled nitric oxide measurement

The role of nitric oxide (NO) in respiratory system pathology has been widely researched. There are conflicting data regarding the precise significance of NO in respiratory illnesses. NO represents a pro-inflammatory factor exhibiting immunomodulatory effects in pathological settings, predisposing to the onset of airway hyperresponsiveness. In physiological circumstances, on the contrary, NO weakly modulates smooth muscle relaxation and protects against airway hyperresponsiveness. Exhaled NO (eNO) is produced by airway epithelial cells. The measurement of NO has the greatest clinical utility in allergic airway disease (56).

Endogenous pulmonary NO production estimated from exhaled air is increased in individuals with cirrhosis and liver failure (43,57-65). A significant negative correlation has been observed between pulmonary vascular resistance and eNO production, indicating that increased NO production may also contribute to cirrhosis-induced pulmonary vasodilatation (57). The eNO concentration has been shown to significantly correlate with the decrease in the alveolar-arterial oxygen gradient (58,59), and the decrease in the eNO concentration following liver transplantation has been found to correlate with the improvement in oxygenation, reinforcing the hypothesis that NO is a key mediator of impaired oxygenation in patients with cirrhosis (63). In addition, it has been reported that there is a definite correlation between the Child-Pugh score (60,61) and NO in exhaled air, and between peak NO concentrations and alkaline phosphatase, aspartate aminotransferase and alanine aminotransferase (ALT), serum albumin and bilirubin (60). Moreover, an increased NO output in exhaled air has been found to correlate with cardiac index, suggesting an association with systemic circulatory impairment in patients with liver cirrhosis (61,62).

In addition, eNO levels have been found to exhibit a negative correlation with DLCO values in patients with liver cirrhosis (43). Furthermore, increased eNO can distinguish individuals with HPS when applying specific cut-offs (63), and has been positively correlated with ascites, portal vein thrombosis, the mucosal red-color sign of varices, and a high hepatic venous pressure gradient (64), and negatively correlated with the average oxygen consumption over 45-60 min of work-time (V'O_2_) peak and a decrease in heart rate reserve, indicating limiting aerobic capacity in patients with liver cirrhosis (65). Of interest, according to a study a low peak exercise oxygen consumption (VO_2_) and reduced eNO may facilitate identifying patients who are at risk to develop perioperative sepsis when undergo liver transplantation (66).

The findings which can be derived from the performance of PFTs in patients with liver cirrhosis are illustrated in Fig. 1, Fig. 2 and Fig. 3. In addition, diagrams of PFTs are illustrated in Fig. S1, Fig. S2, Fig. S3, Fig. S4, Fig. S5 and Fig. S6.

## 9. Pulmonary function testing in children with liver cirrhosis

To the best of our knowledge, only a few studies to date have documented the performance of PFT in children with liver cirrhosis (67-69). As regards spirometry, some researchers have reported obstructive dysfunction (67), others have reported obstructive and/or restrictive ventilatory abnormalities (68), and some studies have found isolated declines in the absolute values of FEV1 and FVC (67,69). FEV1 and FVC values have been observed to be lower in children with HPS compared to those without HPS; however, this difference has not reached statistical significance (69). In addition, alterations in spirometric values have not been related to the duration, histological severity, or grading of fibrosis in children with liver cirrhosis. Decreased DLCO values have been reported in children with liver cirrhosis (67). However, no correlation has been found between a decrease in DLCO values and the duration, histological severity, or grading of fibrosis in children with liver cirrhosis (67).

## 10. Pathogenetic mechanisms for PFT changes in patients with liver cirrhosis

Massive hepatomegaly, ascites, atelectasis and pleural effusions all reduce lung compliance in patients with liver cirrhosis (25). Restrictive dysfunction has been linked to the increasing severity of liver disease and consequences, such as encephalopathy, ascites and hepatic hydrothorax. The mechanism underlying the link between encephalopathy and limitation remains unknown; however, it may be due to the difficulty performing spirometric maneuvers or weakness in the context of end-stage liver disease. Restriction has also been related to lower levels of aminotransferases and albumin (33). The reasons for this are unknown; however, reduced ALT levels in elderly individuals have been linked to frailty and sarcopenia (70). As a result, ALT may be a biomarker of frailty in liver cirrhosis. Restrictive dysfunction is usually associated with ascites and/or pleural effusions, although some patients with restriction abnormalities have neither ascites nor pleural effusions (33). In addition, respiratory muscle weakness is another component that may have contributed to a restrictive dysfunction in these patients (33).

Notably, obesity, systemic inflammation and insulin resistance, all of which have a marked pathophysiological association with diseases responsible for liver cirrhosis, such as NAFLD, can all affect lung function. It has been shown that worsening hepatic steatosis is accompanied by a more rapid loss of lung function. By contrast, an improvement in hepatic steatosis is accompanied by a steady deterioration in pulmonary function, suggesting the presence of a temporal link between changes in fatty liver status and lung function deterioration (71). Obesity, inflammation and insulin resistance are all metabolic risk factors that can affect lung function by activating pulmonary fibrosis or bronchial inflammation and inhibiting airway smooth muscle (72). In a cross-sectional investigation, the homeostasis model assessment of insulin resistance, which represents an indicator of insulin resistance, was found to have an inverse connection with FEV1 and FVC (73). In a previous observational study, individuals with COPD who used antidiabetic medicines that lowered insulin resistance (i.e., insulin sensitizers) had a lower risk of exacerbation of airway inflammation (74).

To date, there have been a few comparable hypotheses, such as insulin resistance causing dysregulation of airway smooth muscle receptors; however, the process by which insulin resistance and lung function degradation are linked is not yet fully understood (75). In addition, a number of inflammatory mechanisms in adipose tissue, skeletal muscle and the liver contribute to insulin resistance development (76). Moreover, serum high-sensitivity C-reactive protein levels have been evaluated as a systemic inflammatory marker linked to decreased lung function (77).

Small airway dysfunction, as indicated by an increased FEF25-75% and an increase in CV, may be due to intrinsic alterations in the small airways, such as muscle edema or muscle spasm, or to changes in the transmural pressure of the airways caused by peribronchial and interstitial edema (18). According to Ruff *et al* (50), the CV in the majority of patients with cirrhosis was higher than predicted, and gas trapping was found in the dependent zone of the lungs. They hypothesized that these abnormalities were caused by interstitial pulmonary edema (50). This is supported by additional evidence provided by laboratory experiments on animals. The small airways in early pulmonary edema models are easily compressed by peribronchial and perivascular cuffing in edema, and there is a significant increase in CV and trapped gas volume in the dependent zone of the lungs (78,79). Interstitial pulmonary edema in liver cirrhosis can be caused by systemic and local factors, such as i) decreased colloid osmotic pressure due to hypoalbuminemia; ii) impairment in lymphatic drainage from the lungs; and iii) an increase in fluid movement from capillaries to interstitial spaces due to increased hydrostatic pressure or altered permeability of the pulmonary capillary membrane due to elevated levels of vasoactive substances (80) and endotoxins (18). Endotoxins can cause hyperdynamic states of circulation in patients with cirrhosis; thus, endotoxins may play a role in the development of interstitial pulmonary edema, leading to small airway dysfunction (18). Intrapulmonary vascular dilatations, widespread interstitial lung illness, pulmonary vaso-occlusive disease, and/or ventilation-perfusion imbalance may all account for gas transfer impairment, as indicated by abnormal DLCO values. An increased capillary plasma volume associated with alveolar capillary dilatation in some patients with severe hepatic disease would be predicted to increase the diffusion distance for carbon monoxide (as well as oxygen) from the alveoli to the red blood cells in the capillary bloodstream, resulting in an increase in the membrane component of diffusion resistance and subsequent hypoxemia (6). Alternative explanations for the decrease in DLCO include early diffuse interstitial lung disease that affects gas exchange, blood flowing through non-ventilated alveoli, anatomic communications between pulmonary arteries and veins that bypass the capillary-alveolar interfaces, and other pulmonary vascular diseases (6).

Although pulmonary hypertension has been linked to cirrhosis and portal hypertension, it is rare, with only a few cases recorded (80,81). Individuals with liver cirrhosis, on the other hand, frequently have low or normal pulmonary vascular resistance (38). Cirrhosis has been shown to be associated with diffuse pulmonary emboli (82) or pulmonary vascular disease with concentric wall thickening of the arteries and veins (83). Even though pulmonary thromboembolism from the portal circulation has been proposed as a cause of pulmonary hypertension in liver disease, Matsubara *et al* (83) failed to demonstrate a statistically significant occurrence of thrombi in the portal and pulmonary vascular beds of patients with hepatic failure. When the restrictive defect is caused by parenchymal pulmonary disease, there is usually a corresponding reduction in DLCO that is disproportionate to the reduction in lung capacity, due to the diffuse parenchymal process involving the microcirculation (6). Interstitial lung disease has been linked to primary biliary cirrhosis (82). There is a considerable link between PBC and Sjogren's syndrome (84), with the latter occurring in half of patients with PBC. Pulmonary function abnormalities linked with Sjogren's syndrome have been thoroughly documented (85), and they include both obstructive and restrictive ventilatory defects. Some researchers have reported that Sjogren's syndrome contributes to the lung abnormalities reported in patients with PBC (86). Chronic active hepatitis has also been linked to interstitial lung disease (87).

The increased alveolar-arterial oxygen gradient seen in liver cirrhosis may be the result of ventilation-perfusion mismatch, right-to-left shunting, or perfusion-diffusion imbalance. Ventilation-perfusion mismatch can occur dur to the following: i) The narrowing and early closure of airways to dependent lung zones due to interstitial edema, pleural effusion, or ascites and/or ii) an imbalance between vasoconstrictor and vasodilator substances that are abnormally metabolized by an impaired liver, with the resultant impairment in hypoxic vasoconstriction leading to relative overperfusion of poorly ventilated (6). Lung ‘spiders’, which are arterial changes in the lungs related to liver cirrhosis, may also contribute to the diffusion abnormalities without restriction, causing right-to-left shunting and/or diffusion-perfusion imbalance (88). Another potential source of the observed oxygenation defect is right-to-left shunting of blood through arteriovenous fistulae, which has been well-described in patients with advanced liver failure and may have accounted for the moderately severe hypoxemia observed in a large proportion of patients with concurrent diffusion abnormalities (88).

The most prevalent acid-base disorder in cirrhotic individuals is the decreased partial pressure of carbon dioxide and respiratory alkalosis (89,90). The precise cause of the aberrant hyperventilation in these patients remains unknown. However, hyperammonemia, ascites, HPS, increased chemosensitivity to CO_2_ and hypoxia, and poor progesterone and estradiol metabolism may all contribute to hyperventilation in individuals with decompensated cirrhosis (90,91). Respiratory muscle weakness and a raised diaphragm due to ascites are two major causes of hyperventilation in individuals with cirrhosis (92). Previous research has linked inspiratory muscle fatigue to higher P0.1 in healthy individuals (93). P0.1 levels have also been found to be elevated in patients suffering from various disorders that induce impaired respiratory muscle strength and dyspnea (94). The impact of inspiratory muscle strength training (IMST) on inspiratory motor drive (P0.1) in healthy volunteers has been investigated, and it has been demonstrated that IMST significantly enhances MIP, which has also been associated with a decrease in P0.1(95). In patients with liver cirrhosis, hyperventilation causes respiratory muscle weakening, which results in increased respiratory motor output and P0.1(15).

Hyperventilation induces the overuse of the respiratory muscles, which may exhibit impairment (35). As regards the lower values of respiratory muscle strength indices in patients with ascites, one possible reason is that these patients have more severe liver disease and a varied degree of mechanical compromise due to ascites (35). Moreover, patients with liver cirrhosis have less muscle mass due to a variety of causes, the most notable of which is protein-calorie deficiency. Another aspect that contributes to muscle mass loss is a decrease in anabolism and an increase in protein catabolism. These nutritional and catabolic consequences on skeletal muscles occur throughout the body, resulting in reduced muscle function in patients with cirrhosis (53).

In individuals with cirrhosis, an increase in portal pressure causes the dilatation of visceral arterial blood vessels throughout the body, as well as a hyperkinetic circulatory condition in combination with portosystemic collateral circulation. Endotoxins and additional gut-produced metabolites can directly stimulate blood vessels or cytokines to derive NO synthase (NOS) and lead to an increased *in vivo* synthesis and release of NO due to decreased liver metabolism, toxin accumulation, increased permeability of the intestinal wall, damaged intestinal motility, and alteration and translocation of the intestinal flora (61). NO is a signaling molecule with a marked involvement in inflammation and tissue damage, and it can widen visceral blood vessels with the elevation of visceral blood flow and aggravate portal hypertension. Increased levels of inflammatory cytokines and endotoxins in the bloodstream of patients with cirrhosis can activate pulmonary vascular endothelial cells to generate NO, which is exhaled outside the body via the respiratory tract (64). An increased NOS expression and elevated NO concentrations in peripheral blood are related to a decrease in the inactivating effects of the liver on endotoxins, and an increase in endotoxin concentrations in the blood circulation in cirrhotic individuals. However, serum NO levels are mostly assessed by detecting its metabolites, nitrate and nitrite, and the results may be incorrect (96). Under the catalysis of NOS, NO is generated *in vivo* from L-arginine and oxygen. Increased NO levels in the pulmonary small airways and alveolar areas of cirrhotic individuals can result in elevation of eNO concentration (58). Excessive eNO production is mostly related to an increase in eNO production by pulmonary vascular endothelial cells, airway epithelial cells and peripheral inflammatory cells (61).

## 11. Alterations in PFTs following specific interventions

Large-volume paracentesis (LVP) is a well-accepted therapeutic option for cirrhotic individuals with tense ascites. Following LVP, the majority of patients experience a symptomatic improvement in breathing (97). The effects of LVP on PFTs in patients with liver cirrhosis have been extensively investigated (20,34,37,97-103). The majority of available studies have reported that LVP results in an increase in lung volumes (34,97-101) and an increase in the values of the spirometric parameters, FEV1, FVC, FEV/FVC, FEF25-75% and PEFR (20,34,37,97-103). As regards DLCO, some studies have demonstrated an increase in its values following LVP (34,100), whereas others have mentioned no change following LVP (97,98,101). Of note, one study on LVP in individuals with tense cirrhotic ascites demonstrated a lack of effect on MIP values, suggesting that the cause is not solely mechanical (102). In addition, the administration of diuretics, such as spironolactone and furosemide has been shown to have positive effects both on the values of spirometric parameters (37,100) and the values of DLCO and lung volumes (100).

Respiratory rehabilitation is an important intervention that has been reported to result in an increase in MIP and MEP values and in the values of FEF25-75% in patients with liver cirrhosis (104). Furthermore, liver transplantation leads to an improvement in arterial oxygenation, but no change in the values of DLCO (105,106). However, liver transplantation results in the normalization of increased eNo values (63).

## 12. Hepatopulmonary syndrome

HPS, which is present in 10-17% of individuals with cirrhosis, is characterized by dilated intrapulmonary vessels, particularly in the basal regions of the lungs. Hypoxemia develops and oxygen therapy may be necessary. Liver transplantation is the sole curative method as it may prevent HPS by closing the shunts. Alveolar-arterial oxygen gradient calculation and contrast echocardiography are two methods used for the diagnosis of HPS. The severity of HPS can be a standalone indication for liver transplantation and is unrelated to the severity of liver disease. Since patients with partial pressure of oxygen levels <50 mmHg and no reversibility to 100% oxygen may be at risk of developing irreversible respiratory failure in the post-transplant period, and having a significant risk of perioperative mortality, it is crucial to accurately identify the severity of HPS (107).

It has been reported that an impaired DLCO is a very common finding when performing PFTs in these patients. DLCO levels have been found to be lower in patients with HPS compared to other patients with cirrhosis, candidates for liver transplantation (108). An impaired DLCO has been described to be independently associated with HPS and has been found to be able to predict the diagnosis of HPS with a considerable discriminative ability (area under the receiver operating characteristic curve, 0.890) (109,110).

## 13. Special considerations

The use of pulmonary PFTs in combination with DLCO to evaluate signs of primary lung disease or the HPS is debatable, and there are significant variations in practice. While some transplantation institutions only screen individuals with symptoms, a history of smoking, or a history of established lung illness, others perform testing to all transplant candidates. More specifically, according to the European Association for the Study of the Liver clinical practice guidelines for liver transplantation candidates, there is a recommendation for performing PFTs to evaluate the respiratory function in all liver transplant candidates (107).

As regards the role of PFTs in the prognosis of patients with liver cirrhosis, as it was mentioned above, DLCO, TLC and RV have been described as significant predictors of ventilator time and both ICU and hospital LOS (P<0.05), but not of patients of graft survival undergoing liver transplantation (38). Moreover, according to another study, although abnormal PFTs are found in a great proportion of patients undergoing liver transplants, they are not associated with complications, graft failure, or mortality following liver transplantation (111).

## 14. Conclusions

Pulmonary complications in patients with liver cirrhosis are accompanied by alterations in PFTs. More specifically, patients with liver cirrhosis present with lower values of the spirometric parameters, FEV1, FVC, FEV1/FVC, PEFR and FEF25-75%, of DLCO, lung volumes, MIP and MEP. In addition, they present with increased values of CV, P0.1 and eNO. These alterations have been shown to be associated with disease severity, clinical features and laboratory parameters in adult patients with liver cirrhosis. Patients with ascites may require therapeutic paracentesis to improve their pulmonary function. Such findings should be considered when assessing patients with liver disease, particularly those who may require surgical intervention. As an impaired lung function and in particular, restrictive dysfunction, can affect post-transplant outcomes such as ventilator time, the hospital LOS and post-operative pulmonary complications, it is critical for the transplant care team to be aware of its prevalence and significance.

## Supplementary Material

Diagram of spirometry. (A) Flow (L/s)-time (sec) Curve. FVC, forced vital capacity; MEF25%, flow at 25% of FVC; MEF 50%, flow at 50% of FVC; MEF75%, flow at 75% of FVC; PEFR, peak expiratory flow rate. (B) Volume-time curve. FEV1, forced expiratory volume in 1 sec; FVC, forced vital capacity; ATS, American Thoracic Society.

Diagram of DLCO. CO, carbon monoxide; CH4, methane; DLCO, diffusion capacity for carbon monoxide; VC, vital capacity.

Diagram of lung volumes. Functional residual capacity (FRC) is the sum of expiratory reserve volume (ERV) and residual volume (RV). Total lung capacity (TLC) is the sum of FRC and inspiratory capacity (IC).

Diagram for MIP and MEP [Pmouth (cmH_2_O)-time (sec) curve]. MIP, maximal inspiratory pressure; MEP, maximal expiratory pressure.

Diagram for single breath gas washout test. Phase I is the very beginning of exhalation where only oxygen is being exhaled and consists primarily of test system and airway deadspace. Phase II is where the gas concentration rises rapidly and consists of mixture of airway and alveolar gas. Phase III is where the gas concentration plateaus and its slope depends on the uniform distribution of gas in the lung. Phase IV is where the gas concentration rises abruptly from the plateau and is considered to be part of the closing volume.

Diagram of P0.1. VT, volume tidal; P0.1, airway occlusion pressure 0.1 sec after the onset of inspiratory flow.

## Figures and Tables

**Figure 1:**
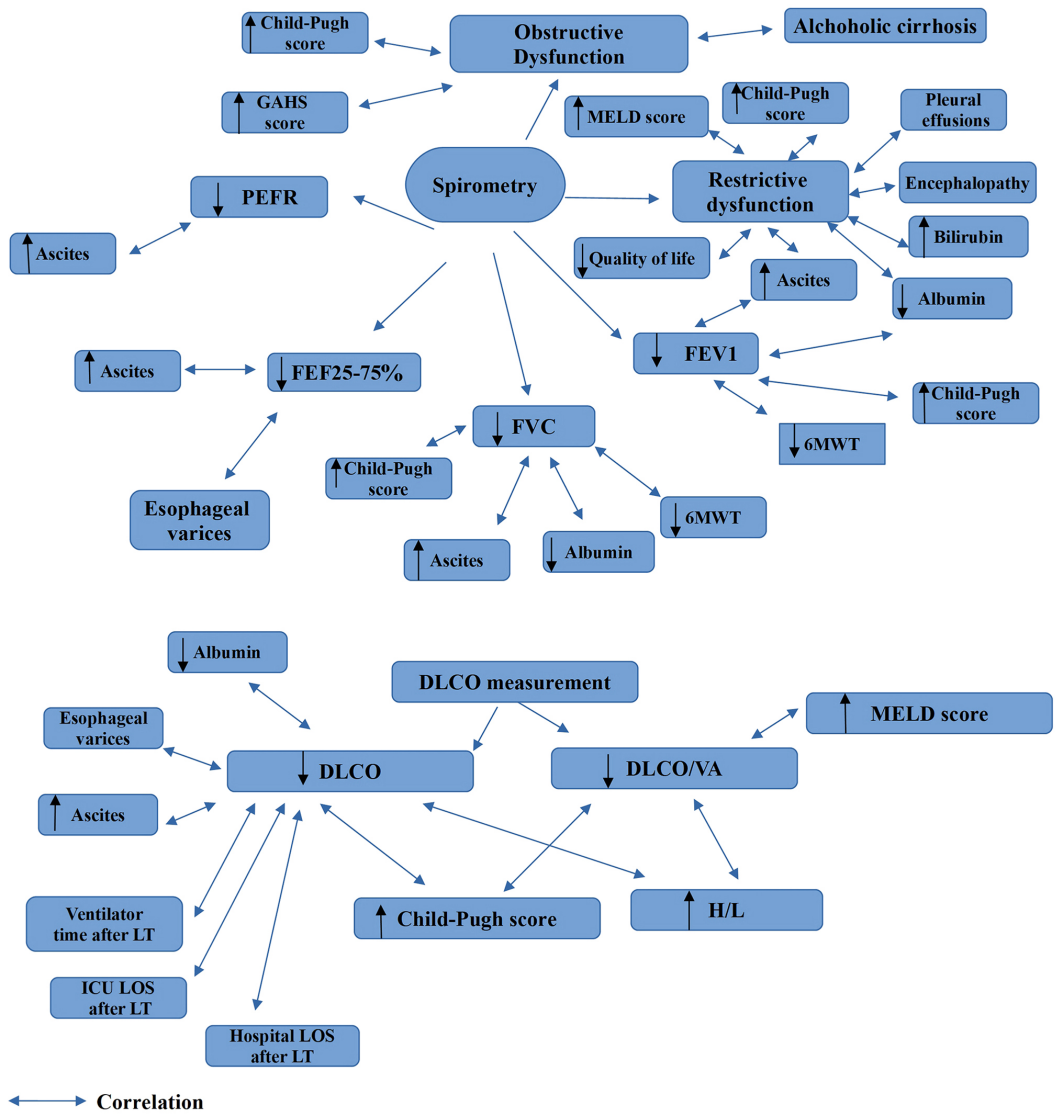
Findings which are obtained from spirometry and DLCO measurements in liver cirrhosis. GAHS, Glasgow alchoholic hepatitis score; DLCO, diffusion capacity for carbon monoxide; H/L, heart liver ratio; FEV1, forced expiratory volume in 1 sec; FVC, forced vital capacity; FEF25-75%, mean forced expired flow as lung volume decreases from 75 to 25% of vital capacity; ICU, intensive care unit; LT, liver transplantation; LOS, length of stay; MELD, Model for End-Stage Liver Disease; PEFR, peak expiratory flow rate; 6MWT, six minute walking test; VA, alveolar volume.

**Figure 2:**
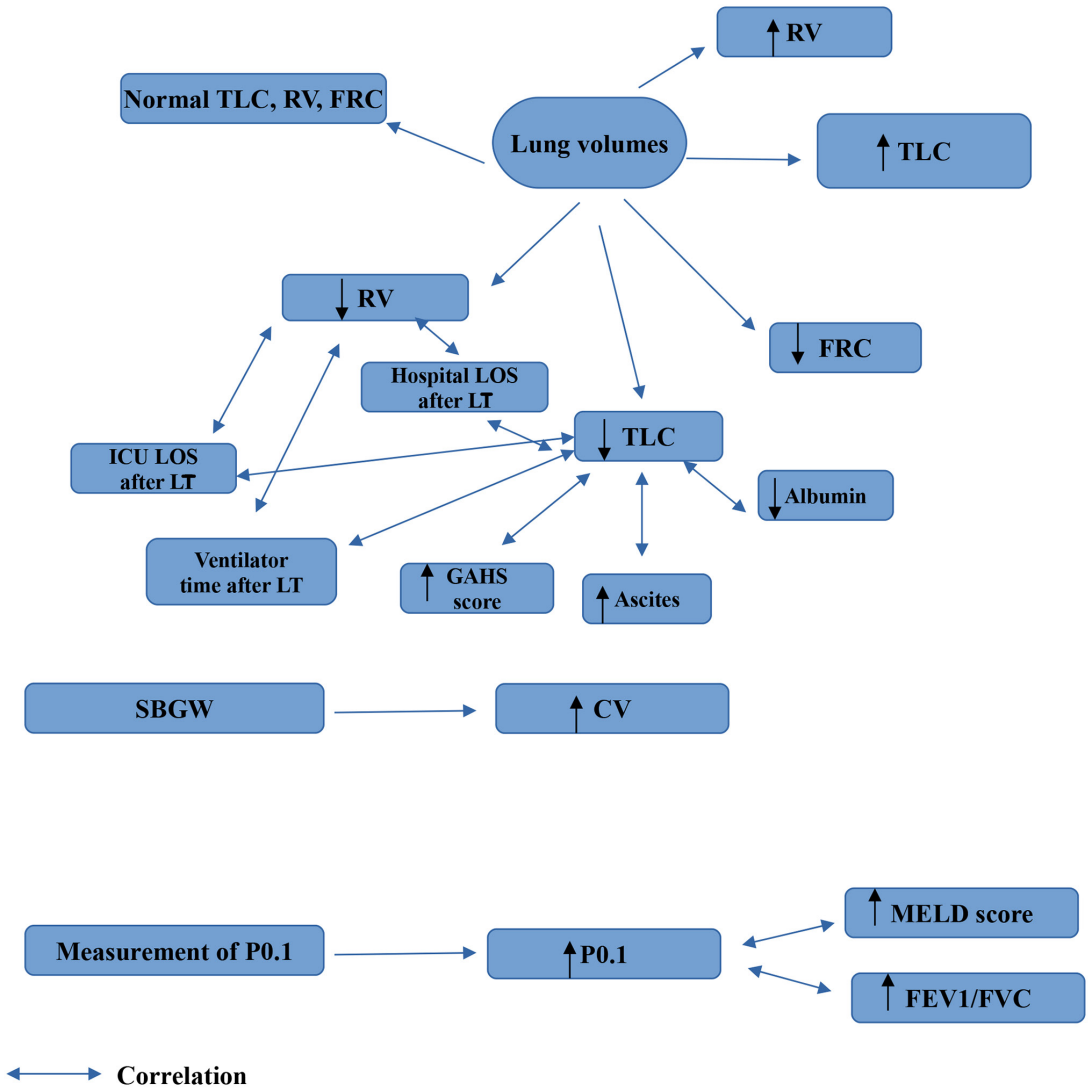
Findings which are obtained from the measurement of lung volumes, SBGW and measurement of P0.1 in liver cirrhosis. CV, closing volume; FEV1, forced expiratory volume in 1 sec; FVC, forced vital capacity; FRC, functional residual capacity; GAHS, Glasgow alchoholic hepatitis score; P0.1, airway occlusion pressure 0.1 sec after the onset of inspiratory flow; RV, residual volume; TLC, total lung capacity; ICU, intensive care unit; LT, liver transplantation; LOS, length of stay; MELD, Model for End-Stage Liver Disease; SBGW, single breath gas washout.

**Figure 3:**
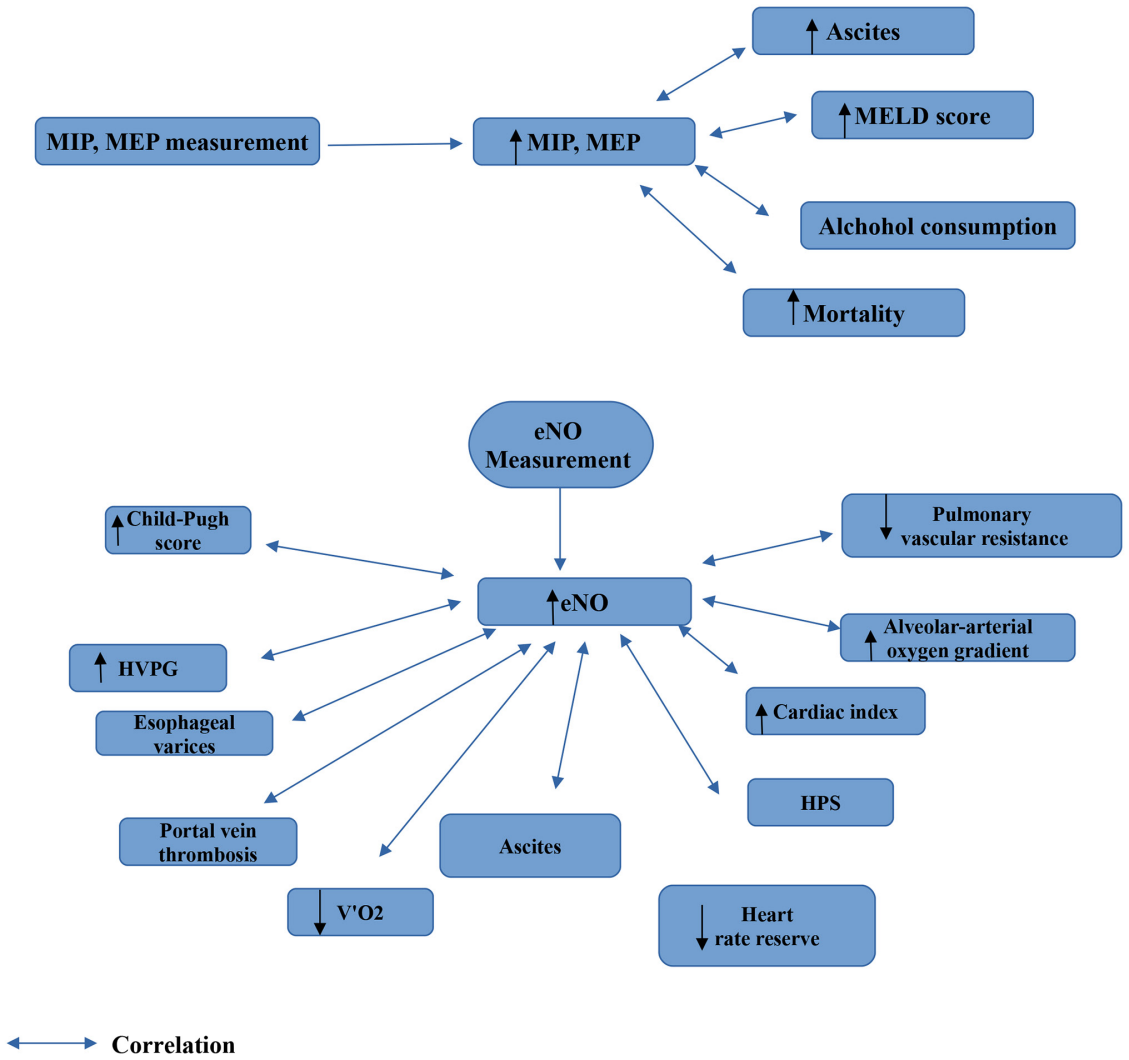
Findings which are obtained from the measurement of MIP, MEP and eNO. eNO, exhaled nitric oxide; MIP, maximal inspiratory pressure; MEP, maximal expiratory pressure; HVPG, hepatic venous pressure gradient; HPS, hepato-pulmonary syndrome; MELD, Model for End-Stage Liver Disease; V'O2, the average oxygen consumption over 45-60 min of work-time.

## Data Availability

Not applicable.
