# Supplementary material for: Pulmonary function testing in patients with liver cirrhosis (Review)
Source: Med Int (Lond). 2023 Jul 6;3(4):36. doi: 10.3892/mi.2023.96 (PMC10391595; doi:10.3892/mi.2023.96)

Figure S1. Diagram of spirometry. (A) Flow (L/s)-time (sec) Curve. FVC, forced vital capacity; MEF25%, flow at 25% of FVC; MEF 50%, flow at 50% of FVC; MEF75%, flow at 75% of FVC; PEFR, peak expiratory flow rate. (B) Volume-time curve. FEV1, forced expiratory volume in 1 sec; FVC, forced vital capacity; ATS, American Thoracic Society.

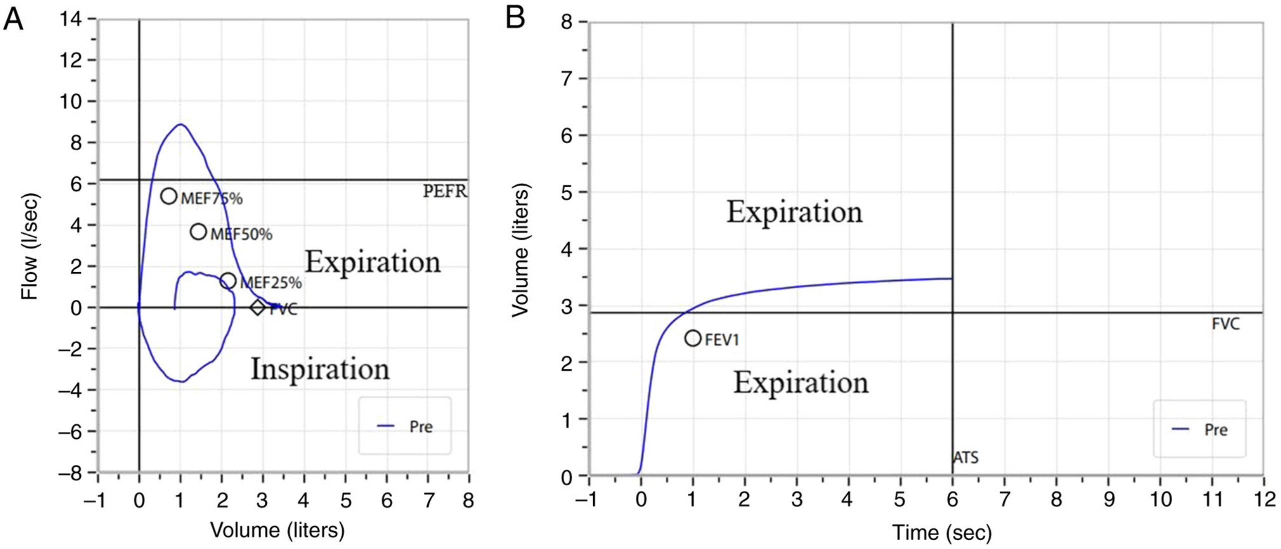

Figure S2. Diagram of DLCO. CO, carbon monoxide; CH4, methane; DLCO, diffusion capacity for carbon monoxide; VC, vital capacity.

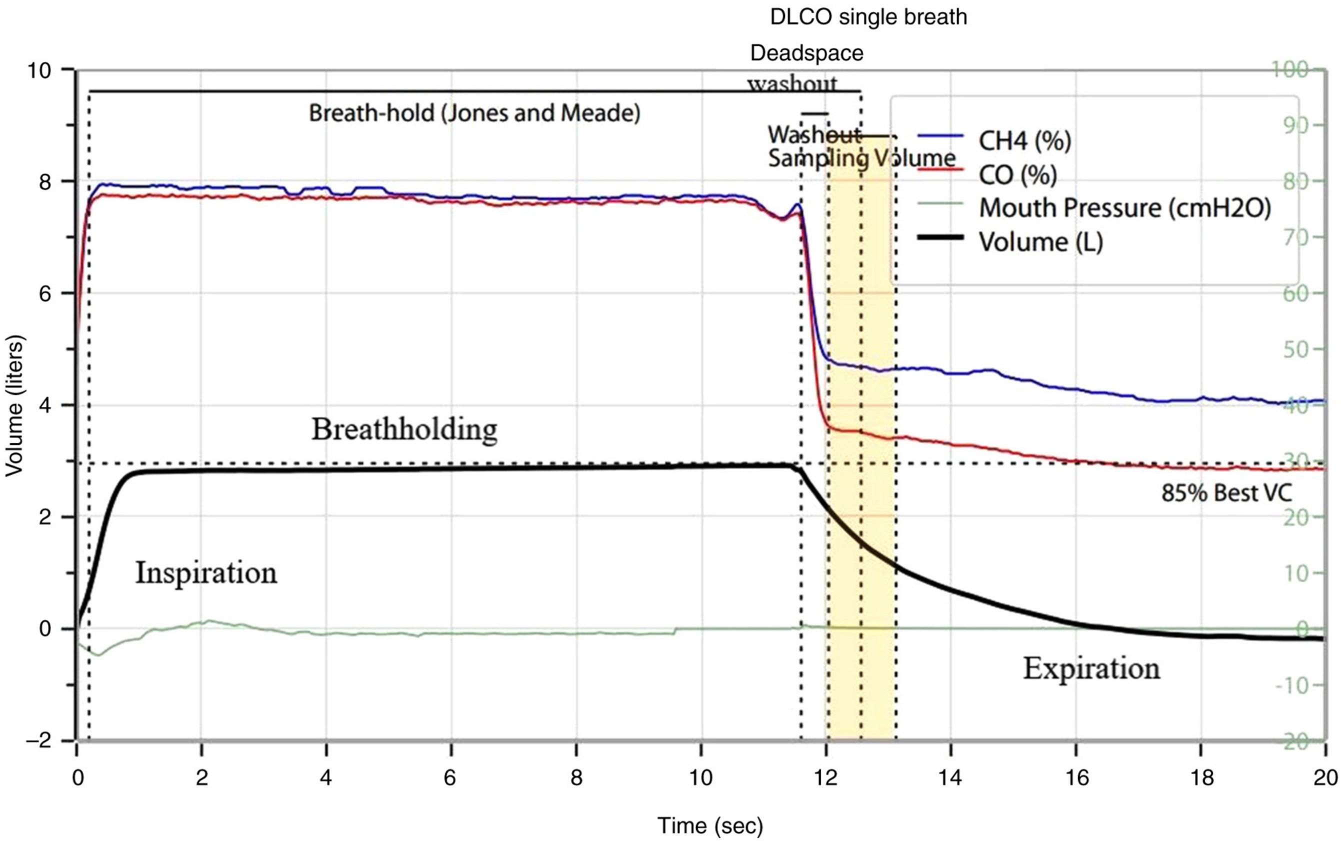

Figure S3. Diagram of lung volumes. Functional residual capacity (FRC) is the sum of expiratory reserve volume (ERV) and residual volume (RV). Total lung capacity (TLC) is the sum of FRC and inspiratory capacity (IC).

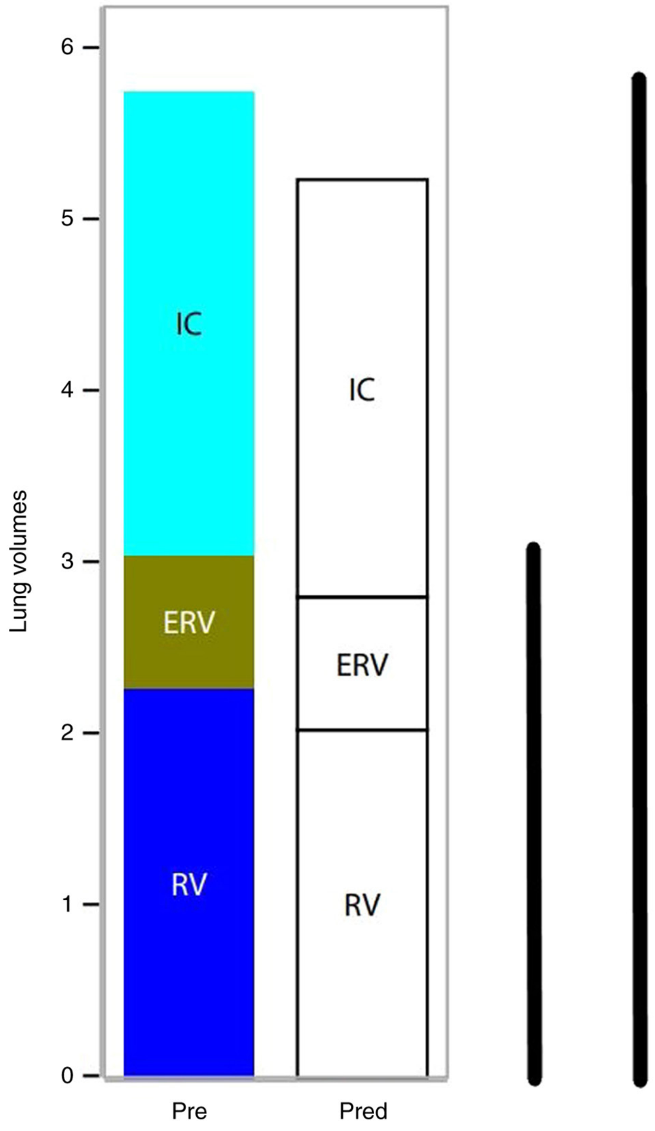

Figure S4. Diagram for MIP and MEP [Pmouth (cmH_2O)-time (sec) curve]. MIP, maximal inspiratory pressure; MEP, maximal expiratory pressure.

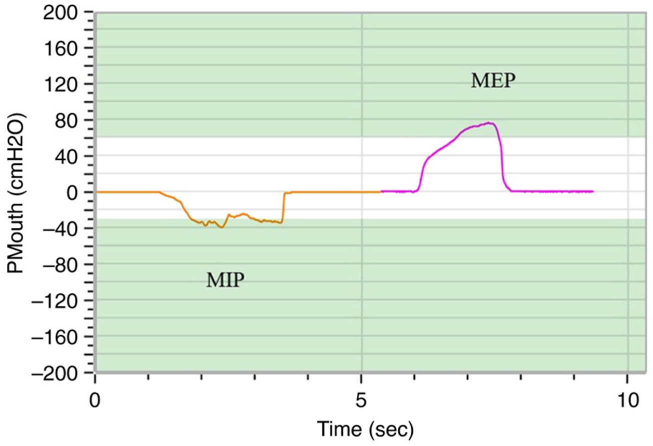

Figure S5. Diagram for single breath gas washout test. Phase I is the very beginning of exhalation where only oxygen is being exhaled and consists primarily of test system and airway deadspace. Phase II is where the gas concentration rises rapidly and consists of mixture of airway and alveolar gas. Phase III is where the gas concentration plateaus and its slope depends on the uniform distribution of gas in the lung. Phase IV is where the gas concentration rises abruptly from the plateau and is considered to be part of the closing volume.

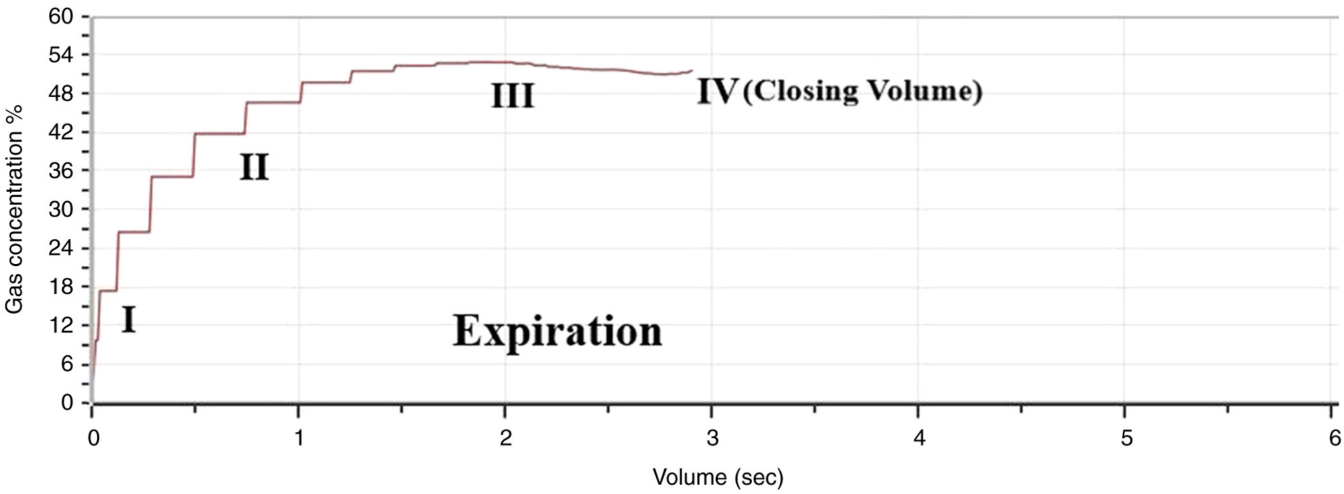

Figure S6. Diagram of P0.1. VT, volume tidal; P0.1, airway occlusion pressure 0.1 sec after the onset of inspiratory flow.

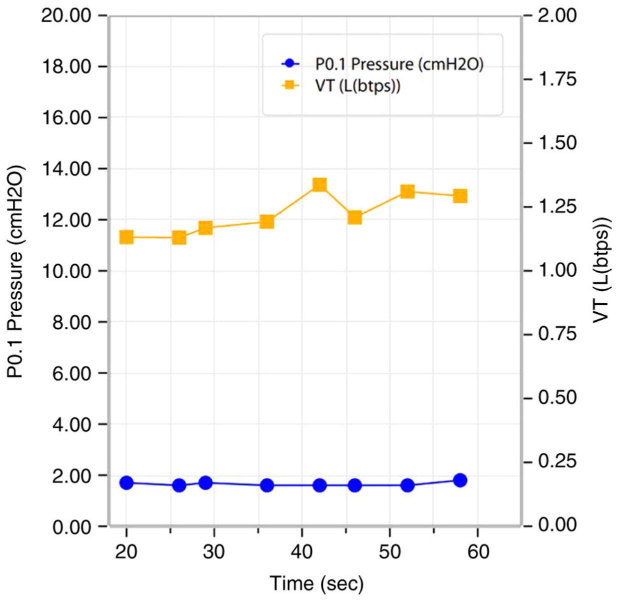

Supplement: Diagram of spirometry. (A) Flow (L/s)-time (sec) Curve. FVC, forced vital capacity; MEF25%, flow at 25% of FVC; MEF 50%, flow at 50% of FVC; MEF75%, flow at 75% of FVC; PEFR, peak expiratory flow rate. (B) Volume-time curve. FEV1, forced expiratory volume in 1 sec; FVC, forced vital capacity; ATS, A [file Supplementary_Data.pdf]
